# Effect of General Anaesthesia Versus Brachial Plexus Block on Neutrophil Count, Neutrophil to Lymphocyte Ratio, and Platelet to Lymphocyte Ratio in Patients Undergoing Upper Limb Fracture Surgeries: A Randomized Clinical Study

**DOI:** 10.7759/cureus.90476

**Published:** 2025-08-19

**Authors:** Usha Shukla, Jay Brijesh Singh Yadav, Shipra Verma, Abhishek Kasaudhan

**Affiliations:** 1 Anaesthesiology and Critical Care, Uttar Pradesh University of Medical Sciences, Saifai, IND; 2 Anaesthesiology, Uttar Pradesh University of Medical Sciences, Saifai, IND

**Keywords:** brachial plexus block, general anaesthesia, immunity, neutrophil, perioperative period, upper extremity

## Abstract

Background: Haematological parameters have emerged as a crucial biomarker for evaluating the immune response and systemic inflammation. This study evaluates the effect of anaesthesia technique on neutrophil count, neutrophil to lymphocyte ratio (NLR), and platelet to lymphocyte ratio (PLR) in upper limb fracture surgeries.

Material and methods: This randomised clinical study was performed on 50 patients of 18 to 60 years of either sex with ASA physical status classification I and II undergoing upper limb fracture surgeries. They were equally divided into two groups: group A (GA) and group B (BPB). Neutrophil count, NLR, and PLR were recorded preoperatively, immediately after surgery, four hours, and 24 hours post-surgery.

Result: NLR was elevated in both groups throughout the postoperative period, with consistently higher values in Group A compared to Group B. The mean neutrophil count (cells/µL) was significantly higher in Group A compared to Group B at both the immediate and four-hour postoperative intervals. Immediately after surgery, the neutrophil count was 5587.86 ± 612.76 in Group A and 5265.64 ± 309.89 in Group B. At 4 hours postoperatively, the count increased to 6248.62 ± 830.49 in Group A and 5578.34 ± 748.05 in Group B (p < 0.05). PLR was lower in Group A than in Group B immediately and four hours after surgery. At 24 hours postoperatively, PLR remained lower than preoperative values in both groups. In Group A, the mean PLR was 176.23 ± 49.10, and in Group B, it was 171.43 ± 62.15. Although the reduction was more pronounced in Group A, the difference was not statistically significant (p >0.05).

Conclusion: This study concluded that GA increases neutrophil count and NLR, while PLR decreases compared to BPB. Adjusting anaesthetic techniques may reduce inflammation and improve recovery.

## Introduction

Surgical trauma triggers a systemic inflammatory response characterized by the release of proinflammatory cytokines, endothelial dysfunction, and neutrophil activation, potentially leading to tissue and organ damage [1]. Various perioperative factors, including the type of anesthesia, blood transfusion, and the extent of surgical tissue injury, significantly influence the immune response [2]. Upper extremity fracture surgeries are commonly performed under general anesthesia (GA) or brachial plexus block (BPB). While GA offers faster induction, it is often associated with undesirable effects such as postoperative nausea, vomiting, drowsiness, and delayed discharge [3]. Conversely, BPB minimizes systemic drug exposure, improves patient satisfaction, and reduces complications like postoperative pain and emesis, making it a preferred technique for upper limb procedures [4,5].

The anaesthetic technique can influence the immune profile. GA, through systemic administration of IV drugs and volatile agents, affects immune function by suppressing phagocytosis and neutrophil chemotaxis. BPB provides localized analgesia without systemic sedation, leading to a reduced physiological stress response and potentially more stable immune parameters [6]. BPB, being a regional anaesthesia, has been shown to contribute to both the anti-inflammatory and immunomodulatory effects, partly due to the inherent property of local anaesthetics and also due to vagal activation as a result of sympathetic blockade [7-9].

Neutrophil count is a direct indicator of acute inflammation and may rise following trauma or infection. Persistent elevation can suggest ongoing inflammation, which may complicate recovery. Recently, systemic inflammatory markers such as the neutrophil-to-lymphocyte ratio (NLR) and platelet-to-lymphocyte ratio (PLR) have gained attention for their prognostic significance. Derived from routine blood tests, these ratios reflect the balance between innate and adaptive immunity [10,11]. Neutrophils, which are rapidly mobilized to surgical sites, play a crucial role in early inflammation, while lymphocytes, as a key in adaptive immunity, often decrease temporarily in response to surgical stress. An elevated NLR postoperatively reflects this immune imbalance, indicating high neutrophil activity and lymphocyte suppression [12-14].

The dynamic interaction between the body’s immunosuppressive mechanisms and inflammatory response during the perioperative period is reflected by fluctuations in NLR and PLR. High NLR is often associated with increased risk of infection, delayed healing, and poor prognosis in various medical and surgical conditions, including cancer and cardiovascular disease [15]. In contrast, lower NLR values may reflect a more favourable immunological state postoperatively. This study aimed to compare the effects of GA against BPB on the NLR as the primary objective, and on the neutrophil count and PLR as the secondary objective.

## Materials and methods

This prospective, randomized, open-label clinical study was carried out from March 2024 to February 2025 at the Uttar Pradesh University of Medical Sciences, Saifai, India, after obtaining approval from the Institutional Ethical Committee with approval number 66/2023-24 on 22/08/2023. This study had been registered in the Clinical Trial Registry of India (CTRI/2024/03/064439). The latest Consolidated Standards of Reporting Trials (CONSORT) guidelines were followed.

On the basis of a previous study conducted by Bengu et al. [16], the sample size was calculated considering an alpha error of 0.05, confidence interval of 95%, power of 80% and taking NLR as the primary variable. Assuming a dropout rate of 10%, the sample size of 25 patients in each group was calculated. All patients were informed thoroughly about both the technique of anaesthesia, their risk-benefit, and written informed consent was obtained. The patients aged between 18 and 60 years of either sex, BMI between 18.5-24.9 kg/m2, American Society of Anaesthesiologists physical status (ASA PS) classification I and II [17], scheduled for upper limb fracture surgeries were included in the study. Patients on chronic treatment with steroids or immunosuppressants, recent chemotherapy, with hepatic and renal dysfunction, patients/attendant refusal, and patients converted to GA due to inadequate BPB were excluded. Computer-generated random number table was used for randomization, and group allocation was concealed using the opaque sealed envelope technique to ensure an equal number of patients in each group. The groups were divided into two: Group A patients received GA, while Group B patients received BPB.

Tablet alprazolam 0.25 mg and tablet ranitidine 150 mg were administered orally on the night prior to surgery. The patient was kept nil per os for 8 hours for solid foods and 2 hours for clear liquids. On the morning of surgery, blood samples were taken in EDTA vials and sent to the laboratory for a complete blood count (CBC) with differential. Total leucocyte count (TLC), neutrophil count, NLR, and PLR were derived from CBC and recorded as baseline values.

In the operation theatre, ASA standard monitoring devices including electrocardiography, non-invasive blood pressure, peripheral oxygen saturation (SpO2) and temperature probe were attached to the patients, and the baseline heart rate (HR), systolic blood pressure (SBP), diastolic blood pressure (DBP), mean arterial pressure (MAP), SpO2 and temperature were recorded. After group allocation, the patients who enrolled in Group A received GA. The patients were premedicated with injection (inj) glycopyrrolate 0.01 mg/kg, inj. midazolam 0.05 mg/kg, and inj. fentanyl 2 µg/kg intravenously (IV), and preoxygenation was done for 3 minutes. The patient was induced with inj. propofol 2 mg/kg IV followed by muscle relaxation with inj. vecuronium 0.1 mg/kg IV. Intubation was performed with a cuffed endotracheal tube of 8 mm internal diameter for males and of 7 mm internal diameter for females. The patient was put on mechanical ventilation. Further anaesthesia was maintained with 50%: 50% oxygen and nitrous oxide, 0.8-1.0% isoflurane, and with intermittent bolus of inj. vecuronium (0.02 mg/kg) IV. At the end of the surgery, muscle relaxation was reversed with inj. neostigmine 0.05 mg/kg IV and inj. glycopyrrolate 0.01mg/kg IV. The patient was extubated after regaining consciousness and demonstrating the ability to follow verbal commands.

Group B patients received ultrasound-guided supraclavicular brachial plexus block using SonoSite M-turbo USG machine (FUJIFILM Sonosite, Bothell, WA) with high frequency (6-13MHz) linear array probe. Under all aseptic conditions, the probe was placed in a coronal oblique plane directly above the midpoint of the clavicle with the patient in a supine position to get a cross-sectional image of the pulsating subclavian artery. The brachial plexus was recognized as a collection of hypoechoic circular structures, located closer to the surface than the subclavian artery. Using a 25-gauge needle, 2 ml of inj. lignocaine 2% was injected into the skin 1 cm lateral to the transducer to decrease the discomfort during needle insertion for the brachial plexus block. A stimuplex needle (B Braun, Melsungen, Germany) 23 gauge, 50 mm was inserted in plane toward the brachial plexus. After careful aspiration, a total volume of 25 ml of local anaesthetic (20 ml of 0.5% bupivacaine, and 5 ml of 0.9% normal saline) was injected by the same needle. 

Surgery was started after adequate anaesthesia was achieved. Hemodynamic parameters were monitored and recorded every 5 min till the completion of surgery in both groups. Immediately after commencing the surgery, the patient had a blood sample drawn and sent for a CBC with differential. Thereafter, CBC with differential was again sent at 4 hours and 24 hours after surgery. From the CBC reports, we derived total leukocyte count, neutrophil count, NLR, and PLR immediately, 4 hours, and 24 hours after surgery.

Data was entered in Microsoft Excel and analysed using statistical software SPSS version 25 (IBM Corp., Armonk, NY). The continuous variables were evaluated by mean (standard deviation). To compare the means between the two or more groups, an analysis by an unpaired Student's t-test was done. Categorial variables were expressed in numbers (percentage) and analysed by a chi-square test.

## Results

**Figure 1:**
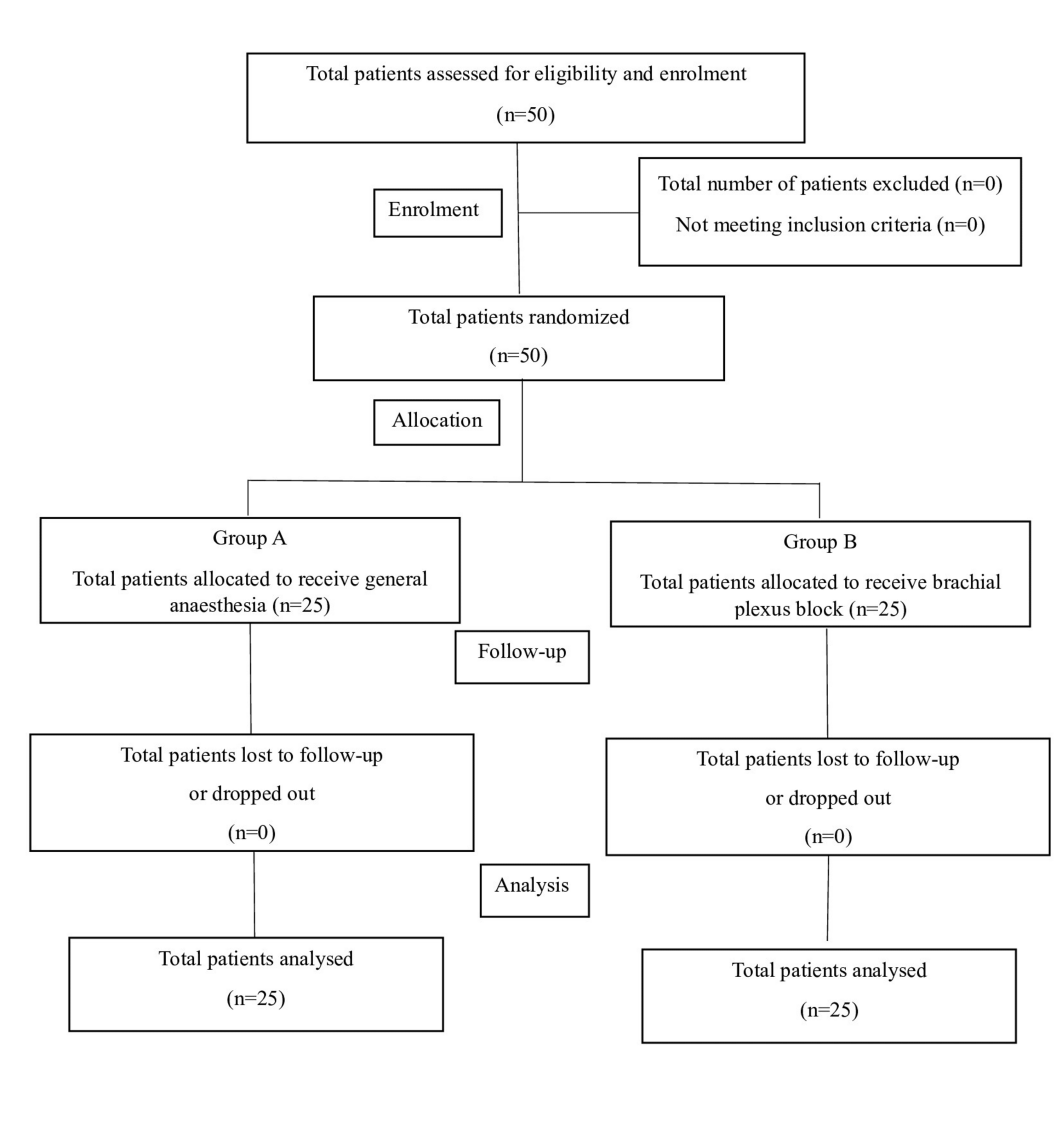
Consolidated Standards of Reporting Trials (CONSORT) flow diagram.

All 50 patients assessed for eligibility were enrolled in the study and underwent randomization. A total of 25 patients in each group received anaesthesia according to group allocation. None of the patients were lost to follow-up or discontinued from the study. All the included patients were analysed (Figure 1). 

The demographic variables were comparable (p > 0.05) between the two groups (Table 1).

**Table 1:** Comparison of demographic profile and patient characteristics. n, number; ASA PS, American Society of Anaesthesiologists Physical Status; data expressed as mean ± standard deviation or as a number; using unpaired t-test for age, weight, BMI, height; chi-square test for gender and ASA PS; P-value >0.05, non-significant.

Parameter			Group A (n=25)	Group B (n=25)	p-value
Age (Years)			38.43 ± 11.71	37.02 ± 10.72	0.659
Gender		Male	18(72.0%)	15(60.0%)	0.550
		Female	7(28.0%)	10(40%)	
Height (cm)			162.79 ± 10.11	164.91 ± 7.66	0. 402
Weight (kg)			73.57 ± 13.55	69.27 ± 11.17	0.227
BMI (kg/m^2^)			21.30 ± 1.89	22.05 ± 1.40	0.117
ASA PS	I		9 (36.0%)	8 (32.0%)	0.800
	II		16 (64.0%)	17 (68.0%)	
Duration of Surgery (minutes)			130.14± 34.56	128.27 ± 29.76	0.838

In the preoperative period baseline TLC count, neutrophil count, NLR, and PLR were comparable in both groups (p > 0.05) (Table 2).

**Table 2:** Comparison of baseline haematological parameters between the groups. n, number; TLC, Total leukocyte count; NLR, Neutrophil-to-Lymphocyte Ratio; PLR, Platelet-to-Lymphocyte Ratio; Data expressed as mean ± standard deviation; Unpaired t-test applied; p value > 0.05, non-significant.

Preoperative Haematological Parameters	Group A (n=25)	Group B (n=25)	p-value
TLC (cells/µL)	7951.93 ± 1879.02	7316.18 ± 1901.45	0.240
Neutrophil Count (cells/µL)	5235.57 ± 762.46	5103.73 ± 1068.89	0.617
NLR	3.12 ± 1.27	3.17 ± 1.13	0.884
PLR	198.67 ± 63.11	202.85 ± 61.00	0.813

The intergroup comparison showed a significantly higher mean TLC in group A compared to group B immediately post-surgery and four hours after surgery. Mean TLC value returned to preoperative value at 24 hours post-surgery. Immediately after surgery, the mean neutrophil count was significantly higher in group A (5587.86 ± 612.76) cells/µL than in group B (5265.64 ± 309.89) cells/µL (p < 0.05). This consistent rise in mean neutrophil count was highest in group A (6248.62 ± 830.49) cells/µL compared to group B (5578.34 ± 748.05) cells/µL at 4 hours after surgery, which was statistically significant (p < 0.05). At 24 hours post-surgery mean neutrophil count was also significantly higher in group A than in group B (Table 3).

**Table 3:** Comparison of postoperative haematological parameters between the groups. n, number; TLC, Total leukocyte count; NLR, neutrophil-to-lymphocyte ratio; PLR, platelet-to-lymphocyte ratio; Data expressed as mean ± standard deviation; Unpaired t-test applied; p value< 0.05 significant; p value < 0.001 highly significant.

Postoperative Haematological Parameters	Group A (n=25)	Group B (n=25)	p-value
Immediately after surgery			
TLC (cells/µL)	8617.64 ± 2140.28	6873.91 ± 1738.51	< 0.05
Neutrophil count (cells/µL)	5587.86 ± 612.76	5265.64 ± 309.89	< 0.05
NLR	6.27 ± 1.38	4.23 ± 1.02	< 0.001
PLR	148.44± 40.87	178.61 ± 57.83	< 0.05
4 Hours after surgery			
TLC (cells/µL)	8227.00 ± 1265.78	7552.64 ± 957.13	< 0.05
Neutrophil count (cells/µL)	6248.62 ± 830.49	5578.34 ± 748.05	< 0.05
NLR	5.39 ± 1.21	3.93 ± 1.20	< 0.001
PLR	128.95± 40.10	163.47 ± 75.05	< 0.05
24 Hours after surgery			
TLC (cells/µL)	7204.00 ± 2216.37	7373.00 ± 2616.16	0.806
Neutrophil count (cells/µL)	5792.68 ± 667.11	5432.68 ± 585.72	< 0.05
NLR	4.87 ± 1.16	3.42 ± 1.21	< 0.001
PLR	176.232 ± 49.10	171.43 ± 62.15	0.751

The mean NLR was comparable in both groups at baseline, and it increased in both groups postoperatively. Change in mean NLR was significantly higher in group A (6.27 ± 1.38) than in group B (4.23 ± 1.02) immediately after surgery (p < 0.001). These changes in mean NLR were consistent with higher values in group A than group B at 4 hours and 24 hours after surgery, as shown in Table 3. Change in mean PLR was significantly lower in group A than group B immediately after surgery (148.44± 40.87 and 178.61 ± 57.83, respectively, p < 0.05), and 4 hours after surgery (128.95± 40.10, and 163.47 ± 75.05, respectively, p < 0.05). No significant difference was seen in mean PLR in both groups at 24 hours after surgery (Table 3). 

**Figure 2:**
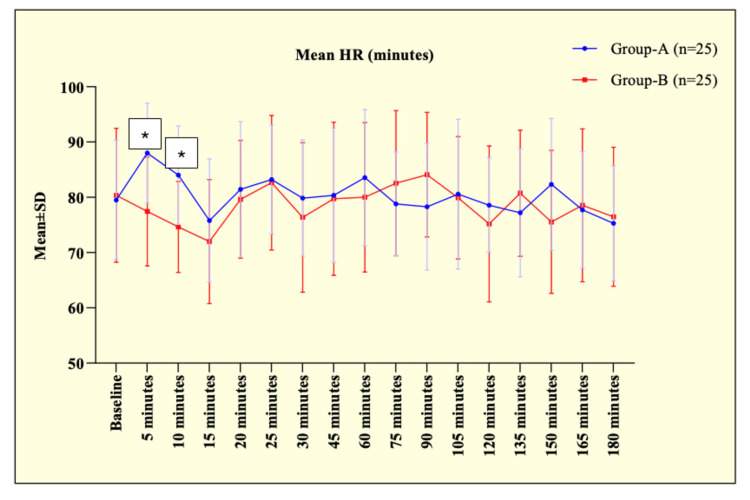
Line diagram showing comparison of mean HR at different time interval between the groups. * p-value < 0.05, significant

**Figure 3:**
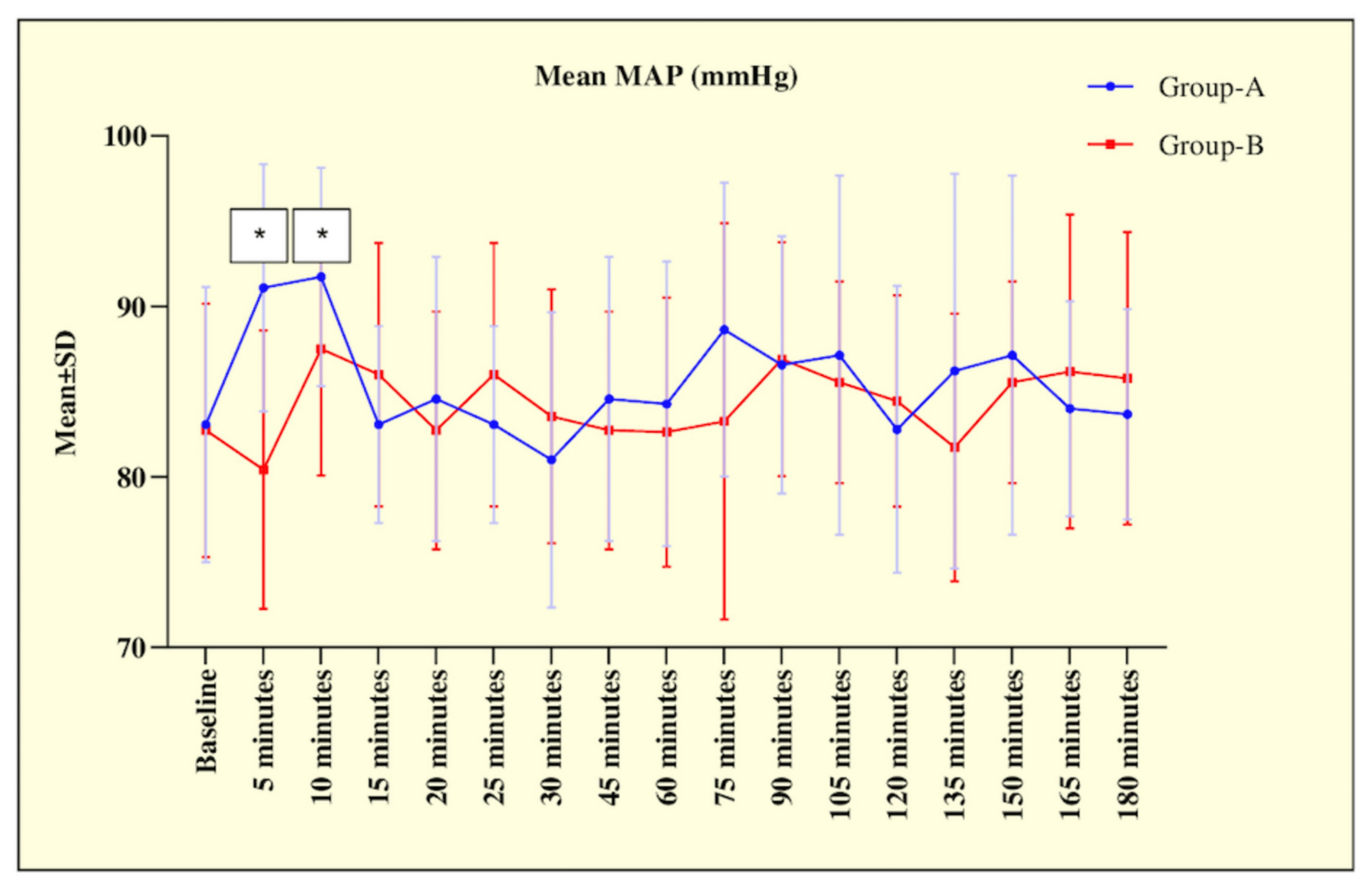
Line diagram showing comparison of mean MAP at different time interval between the groups. * p-value < 0.05, significant

The hemodynamic parameters HR and MAP were comparable preoperatively before the start of interventions in both groups. However, at five minutes, patients in group A had a significantly higher HR (88.00 ± 9.04) bpm and MAP (91.09 ± 7.25) mmHg compared to group B (77.45 ± 9.86) bpm, (80.43 ± 8.16) mmHg, respectively (p < 0.001), suggesting a notable early rise of HR and MAP in patients underwent surgery under GA and a similar significant difference persisted till 10 minutes as shown in Figures 2-3.

This could be due to the stress response of laryngoscopy and intubation in group A, whereas group B maintained a more stable haemodynamic profile.

## Discussion

This study was conducted with the aim of comparing the effect of GA and BPB on neutrophil count, NLR, and PLR in patients undergoing upper limb fracture surgeries. In upper limb fracture surgeries, BPB either by itself or in conjunction with GA, is frequently utilized. By doing this, the stress reaction to surgery can be reduced. Neutrophil count, NLR, and PLR are essential markers to give insight into inflammatory response [18]. Postoperatively, delayed immune response might occur due to a variety of conditions, including the type of surgery and anaesthetic technique used. The NLR as it is simple, easy to procure, and inexpensive, can be an option to look for the inflammatory response in the patients, so this study was an attempt to answer some of these queries to a large extent for the better outcome of the patient. In this study, BPB is found to be better with a significantly less change in neutrophil count, NLR, and PLR compared with those who received GA for upper limb fracture surgeries. Similarly, Netra et al., evaluated the relationship between NLR and anaesthetic techniques in patients undergoing infraumbilical surgeries and concluded that postoperative NLR in these patients after spinal anaesthesia was significantly low as compared to that after general anaesthesia [19].

In concordance with our study, Bengu. et al. [16] had also evaluated the impact of anaesthesia techniques on inflammatory markers in patients undergoing forearm surgery. They found that the increase in NLR during the postoperative period was significantly higher in the GA group compared to the BPB (p < 0.001) [16]. These findings suggest that peripheral nerve blocks may play a role in reducing inflammation and alleviating stress response compared to GA.

Local anaesthetics used in BPB possess direct anti-inflammatory properties, inhibiting neutrophil migration and reducing the release of pro-inflammatory cytokines, thereby mitigating the postoperative inflammatory response. BPB also provides superior postoperative analgesia, resulting in less pain and a reduced pain-induced neuroendocrine stress response, which in turn prevents the cortisol-driven mobilisation of neutrophils into circulation [20]. The aforesaid are the possible reasons for less rise in NLR in patients who received BPB at all time points postoperatively compared to the patients who received GA. At 24 hours of surgery, NLR was normalised to baseline value in BPB, reflecting resolution of acute stress response as the body returns to immunological homeostasis in the absence of further insult, while in the GA group it still remains significantly high.

This increase in neutrophil count and NLR in group A is largely part of the stress response triggered by GA, involving the activation of the hypothalamic-pituitary-adrenal (HPA) axis and release of catecholamines and cortisol. These hormones mobilize neutrophils from the marginal pool into the bloodstream, contributing to a higher mean neutrophil count and NLR [21]. Despite the increased neutrophil count, the functioning of neutrophils may be impaired under GA, causing reduced chemotaxis, impaired phagocytosis. These effects are transient but can compromise early immune defence mechanisms, increasing the risk of postoperative infection, especially in major surgeries or in immunocompromised patients. One of the previous studies conducted by Boruah et al., [22] also supported our study results, in which they compared the effect of three different anaesthetic techniques [GA versus total intravenous anaesthesia (TIVA) versus combined spinal epidural anaesthesia (CSE)] on NLR. They demonstrated an increase in NLR in all three anaesthesia techniques, and the increase in mean NLR was highest in GA at the end of the surgery, 6 hours, and 24 hours after surgery. However, the difference in change in NLR was found to be insignificant when comparison was made between TIVA and CSE. Use of multiple anaesthetic agents and mechanical ventilation, as seen in general anaesthesia, can trigger additional inflammatory responses, which are avoided in brachial plexus block, contributing to less rise in TLC, neutrophil count, and NLR.

In our study, PLR decreases immediately and at 4 hours postoperatively, which might be due to haemodilution and platelet consumption at the surgical site following activation of the clotting cascade. Within 24 hours, PLR tends to return to normal as platelet regeneration begins.

There are some limitations to the present study. First, although the sample size was relatively small compared to similar studies, it remains modest, which may limit the generalizability of our findings. Another important limitation is that the haematological parameters were monitored only up to 24 hours postoperatively. This relatively short follow-up period may not be sufficient to fully understand the trajectory of immune changes or any complications related to anaesthesia type. Furthermore, clinical outcomes such as the presence of surgical site or systemic infections beyond 24 hours, which might be influenced by the anaesthesia technique, were not assessed. Inflammatory markers such as C-reactive protein, erythrocyte sedimentation rate, and procalcitonin, despite being widely available and clinically relevant, which could provide deeper insight into the immune response, were not explored in our study. We could not evaluate correlations between TLC, neutrophil count, NLR, PLR, and the incidence of postoperative infections. Further studies can be planned to overcome these limitations.

## Conclusions

This study concluded that the choice of anaesthesia technique can influence the postoperative immune response, as evidenced by changes in haematological markers such as TLC, neutrophil count, NLR, and PLR in patients undergoing upper limb fracture surgeries. It has been seen that the patient who received GA had an increase in TLC, neutrophil count, NLR, and a decrease in PLR at different time intervals than BPB. So, according to this study, it can be concluded that anaesthesia techniques can be modified for patients undergoing upper limb fracture surgeries in order to reduce the postoperative inflammatory response. Haematological inflammatory markers like TLC, neutrophil count, NLR, and PLR can be considered as simple, cost-effective markers to check the anaesthesia-induced inflammatory response in the postoperative period.
